# Dental service patterns among private and public adult patients in Australia

**DOI:** 10.1186/1472-6963-8-1

**Published:** 2008-01-03

**Authors:** David S Brennan, Liana Luzzi, Kaye F Roberts-Thomson

**Affiliations:** 1 Australian Research Centre for Population Oral Health, School of Dentistry, The University of Adelaide, Adelaide, South Australia 5005, Australia

## Abstract

**Background:**

While the majority of dental care in Australia is provided in the private sector those patients who attend for public care remain a public health focus due to their socioeconomic disadvantage. The aims of this study were to compare dental service profiles provided to patients at private and public clinics, controlling for age, sex, reason for visit and income.

**Methods:**

Data were collected in 2004–06, using a three-stage, stratified clustered sample of Australians aged 15+ years, involving a computer-assisted telephone interview (CATI), oral examination and mailed questionnaire. Analysis was restricted to those who responded to the CATI.

**Results:**

A total of 14,123 adults responded to the CATI (49% response) of whom 5,505 (44% of those interviewed) agreed to undergo an oral epidemiological examination. Multivariate analysis controlling for age, sex, reason for visit and income showed that persons attending public clinics had higher odds [Odds ratio, 95%CI] of extraction (1.69, 1.26–2.28), but lower odds of receiving oral prophylaxis (0.50, 0.38–0.66) and crown/bridge services (0.34, 0.13–0.91) compared to the reference category of private clinics.

**Conclusion:**

Socio-economically disadvantaged persons who face barriers to accessing dental care in the private sector suffer further oral health disadvantage from a pattern of services received at public clinics that has more emphasis on extraction of teeth and less emphasis on preventive and maintenance care.

## Background

The majority of dental care in Australia is provided in the private sector, with approximately 85% of dentists working in private practice [1]. While patients who attend for public dental care are a minority they remain a public health focus due to their socioeconomic disadvantage. Public dental patients must generally be health care card holders in order to be eligible for public dental care. Health cards are issued by the Australian government to eligible persons on pensions and allowances such as unemployed persons and aged pensioners. These people comprise a low income group, who by definition are socio-economically disadvantaged.

Previous reports have shown that card holders attending for dental care are at disadvantage in terms of access to care, typically having to spend time on long waiting lists before receiving treatment or accessing limited emergency care in the short term [2]. Care received by public dental patients has been characterised by high levels of emergency care and high rates of tooth extraction [3,4]. A government program designed to ameliorate this disadvantage was implemented between 1994 and 1996 [5]. However, since then there had been a reported deterioration in oral health status among public dental patients [6].

Reports continue to show that a substantial minority of the population suffer affordability and hardship issues in relation to accessing dental care [7]. Introduction of government funded private insurance subsidy schemes have been shown to favour higher income groups [8]. Previous reports of service patterns were based on separate studies of private sector [9,10] and public sector patients [3,4], which hindered comparability. To better contrast private and public service patterns a single study was needed where comparisons can be made using the same measures in order to eliminate potential methodological biases. The aims of this study were to utilise a single study in order to compare dental service profiles provided to patients at private and public clinics, controlling for age, sex, reason for visit and income.

## Methods

### Sampling and data collection

The 2004–06 National Survey of Adult Oral Health (NSAOH) involved a three-stage, stratified clustered sampling design to select a sample of Australians aged 15+ years from households with listed telephone numbers in an electronic white pages (EWP) database [11]. From this sampling frame 15 strata were selected, with population proportional to size selection. The strata comprised metropolitan and non-metropolitan areas of 7 states/territories and the single stratum of the Australian Capital Territory. Postcode comprised the primary sampling unit, with household being the secondary sampling unit. Postcodes represented the geographic clustering in the design and were selected with probability proportional to size, where size was defined as the number of households listed in the EWP database in each postcode. The second stage of sampling selected a systematic sample of households listed in the EWP database for each sampled postcode. Thirty households per metropolitan stratum and 40 households per non-metropolitan stratum were selected, after elimination of non-residential phone numbers identified during initial contact by telephone interviewers. The sample was approached to participate in a computer-assisted telephone interview (CATI) where the final stage of sampling entailed the random selection of one person aged 15 years or more per household, followed by an oral epidemiological examination and a mailed questionnaire.

Approximately 10 days prior to dialling each sampled telephone number, a primary approach letter explaining the purpose of the survey was mailed to the address that accompanied each sampled telephone number. On each occasion when interviewers dialled a sampled telephone number, a record was made on the computer system. In the CATI, interviewers read questions from a computer screen and recorded answers directly onto the computer. The interview comprised 79 questions, with skip sequences built into the CATI computer system so that questions flowed without intervention from the interviewers. Each sampled telephone number was called up to 6 times at varying times of day and evening, and on different days of the week. Where there was no answer after 6 calls, the number was abandoned and recorded as a 'non-contact'. When a sampled person was identified up to 6 additional calls were made in an attempt to contact them. If the target person did not speak English an attempt was made to conduct a proxy interview with a resident of the household who spoke English, and in some instances interviews were conducted in foreign languages. People who reported having some or all of their own natural teeth were invited to attend an examination, after which they were sent a mailed self-complete questionnaire.

### Variables measured

In the first stage of data collection respondents supplied information during a CATI on variables such as self-reported health status, use of dental services, demographics and socio-economic status. The percentage of persons who reported receiving dental services in the categories of fillings, extractions, oral prophylaxis, x-rays, crown/bridge, dentures and gum treatment within the last year comprised the dependent variables. The explanatory variables consisted of site of last visit, sex, reason for last visit, income, and age. At the examination, clinical oral status was collected from dentate people by dentists trained in standard survey procedures [11], but these data are not used in this analysis. Further information, such as psycho-social variables, also not used in this analysis, was collected later through a mailed questionnaire. The research was approved by the Human Research Ethics Committee of the University of Adelaide.

### Analysis

The analysis was restricted to dentate persons aged 15 years or older, who had made a dental visit within the last year. Data were weighted by state/territory, metropolitan/non-metropolitan location, age and sex. To account for design effects associated with the complex sample design, data were analysed using survey procedures that adjusted for strata and primary sampling units [12]. The percentages presented in Tables 1 and 2 are weighted while the cell numbers are unweighted. Initially age-specific distributions of the explanatory variables of site of last visit, sex, reason for last visit, and income were tabulated (note that these weighted percentages may differ from that obtained by using the unweighted numbers of respondents as the denominator). Unadjusted bivariate associations of this set of explanatory variables were then tabulated for the percentage of persons receiving dental services in the last year. The percentages in Table 2 express each cell total as a percentage of the row total. For example, consider the 41.7% (weighted %) of persons who last visited privately and who reported receiving 1 or more fillings. This weighted cell percentage was calculated based on weighted counts and was obtained by dividing the number of persons who last visited privately and reported receiving 1+ fillings from Table 2 by the total number of persons who last visited privately from Table 1 (ie, the corresponding unweighted percentage would be obtained by dividing the unweighted n = 3,046 from Table 2 by the unweighted n = 7,011 from Table 1). Adjusted odds ratios were then determined from multivariate logistic regression models of persons receiving dental services in the last year, with the dependent variables coded as 1 if a service was received with the reference category of no service received coded as 0. In the bivariate analysis and multivariate models site of last visit was restricted to private and public clinics as this was the contrast of central interest to the aims, and due to relatively small cell sizes available for other sites of last visit (ie, school, technician, other).

**Table 1:** Distribution of explanatory variables

	**AGE CATEGORY (col%)**														
	**15–34 years**			**35–44 years**			**55–74 years**			**75+ years**			**All ages**		
	n^(a)^	%^(b)^	SE^#^	n^(a)^	%^(b)^	SE^#^	n^(a)^	%^(b)^	SE^#^	n^(a)^	%^(b)^	SE^#^	n^(a)^	%^(b)^	SE^#^
**Site of last visit**^(p ≤ 0.0001)^															
Private	1479	80.8	1.2	2959	90.4	0.7	2086	87.6	0.9	487	90.5	1.4	7011	86.5	0.6
Public	166	9.0	0.9	197	5.9	0.6	234	8.9	0.7	54	8.2	1.3	651	7.7	0.5
School	71	4.2	0.6		0.0	0.0	1	0.0	0.0		0.0	0.0	72	1.4	0.2
Technician	1	0.0	0.0	12	0.4	0.1	15	0.7	0.2	1	0.2	0.2	29	0.3	0.1
Other	97	6.0	0.8	108	3.4	0.4	58	2.8	0.4	5	1.1	0.5	268	4.0	0.3
**Sex**^(ns, p = 0.230)^															
Male	674	47.8	1.5	1203	47.2	1.1	953	49.1	1.2	216	42.6	2.3	3046	47.6	0.8
Female	1147	52.2	1.5	2073	52.8	1.1	1442	50.9	1.2	331	57.4	2.3	4993	52.4	0.8
**Last visit**^(p ≤ 0.0001)^															
Check-up	1132	65.1	1.4	1721	53.5	1.1	1204	50.6	1.4	319	58.4	2.5	4376	57.0	0.8
Problem	689	34.9	1.4	1554	46.5	1.1	1191	49.4	1.4	227	41.6	2.5	3661	43.0	0.8
**Income**^(p ≤ 0.0001)^															
<$20 K	103	5.6	0.7	251	6.4	0.5	574	22.1	1.1	254	46.5	2.8	1182	12.1	0.5
$20–<40 K	274	17.3	1.3	514	15.0	0.8	699	30.2	1.1	149	33.4	2.5	1636	20.3	0.6
$40–<60 K	335	20.5	1.4	657	20.4	0.9	369	17.0	0.9	50	11.9	1.9	1411	19.2	0.6
$60–<80 K	281	20.1	1.4	593	19.2	0.9	209	10.6	0.8	21	4.8	1.3	1104	16.6	0.6
$80+K	499	36.6	1.7	1107	39.0	1.3	346	20.1	1.3	14	3.4	1.3	1966	31.9	0.9
															
**ALL (row%)**	1821	33.8	0.8	3276	38.1	0.7	2395	22.4	0.7	547	5.7	0.3	8039	100.0	0.0

(a) unweighted

(b) weighted

# Standard errors corrected for complex sample design

**Table 2:** Bivariates: % receiving 1+ services by explanatory variables

	**Services received (cell%)**																				
	
	**Fillings**			**Extractions**			**Oral prophylaxis**			**X-rays**			**Crown/Bridge**			**Dentures**			**Gum treatment**		
	n^(a)^	%^(b)^	SE^#^	n^(a)^	%^(b)^	SE^#^	n^(a)^	%^(b)^	SE^#^	n^(a)^	%^(b)^	SE^#^	n^(a)^	%^(b)^	SE^#^	n^(a)^	%^(b)^	SE^#^	n^(a)^	%^(b)^	SE^#^
**Site of last visit**					**			**			**			**			**				
Private	3046	41.7	0.8	896	13.0	0.5	5156	72.9	0.8	3224	45.7	0.8	543	7.0	0.4	231	2.9	0.2	363	4.5	0.3
Public	317	47.0	2.7	200	30.6	2.4	287	43.7	2.6	310	51.7	2.8	12	2.1	0.9	58	6.0	0.9	28	4.6	1.2
**Sex**					**			**													
Male	1334	42.7	1.2	486	16.2	0.9	2054	67.8	1.2	1420	46.7	1.2	232	6.6	0.6	121	3.2	0.3	137	3.9	0.4
Female	2176	41.3	0.9	657	12.8	0.6	3645	72.8	0.9	2275	45.7	1.0	344	6.4	0.4	201	3.3	0.3	268	4.8	0.4
**Last visit**		**			**			**			**			**			**			**	
Check-up	1271	28.1	0.9	225	5.8	0.5	3587	80.0	0.8	1540	34.8	0.9	158	3.1	0.3	85	1.6	0.2	190	3.6	0.3
Problem	2239	60.5	1.1	918	25.9	0.9	2111	57.7	1.2	2154	61.4	1.1	417	11.0	0.7	237	5.4	0.4	214	5.4	0.5
**Income**		**			**			**			**			**			**				
<$20 K	533	44.6	1.9	262	22.9	1.5	702	58.9	1.7	460	38.9	1.8	47	4.1	0.7	124	9.6	1.0	62	5.4	0.9
$20–<40 K	784	47.0	1.5	288	19.1	1.1	1120	65.8	1.6	730	46.7	1.6	118	6.8	0.7	82	4.1	0.6	89	4.4	0.6
$40–<60 K	658	45.7	1.6	209	16.6	1.3	999	70.0	1.5	705	50.5	1.7	113	7.6	0.9	37	2.5	0.5	76	4.8	0.7
$60–<80 K	477	41.9	1.9	113	10.9	1.2	819	72.2	1.8	516	45.6	1.9	71	5.5	0.8	22	2.1	0.6	44	4.0	0.8
$80+K	765	38.7	1.4	187	9.3	0.9	1564	79.0	1.3	965	47.5	1.4	175	7.8	0.7	36	2.1	0.4	107	4.7	0.6
**Age group**		**			**			**			**			**			**			**	
15–34 years	653	34.5	1.4	261	14.1	1.1	1180	65.6	1.4	835	45.4	1.5	52	2.9	0.5	10	0.5	0.2	54	2.5	0.5
35–54 years	1453	44.7	1.1	442	14.4	0.7	2354	72.2	1.0	1614	49.0	1.1	255	7.5	0.5	87	2.6	0.3	185	5.1	0.5
55–74 years	1150	47.5	1.3	370	15.0	0.9	1758	74.0	1.1	1061	45.3	1.3	230	10.1	0.8	170	6.7	0.6	143	6.1	0.6
75+ years	254	47.0	2.7	70	14.8	1.9	407	73.1	2.3	185	35.6	2.4	39	7.3	1.3	55	10.5	1.6	23	4.6	1.1
																					
**ALL**	3510	42.0	0.7	1143	14.5	0.5	5699	70.4	0.8	3695	46.2	0.7	576	6.5	0.3	322	3.3	0.2	405	4.4	0.3

(a) unweighted

(b) weighted

# Standard errors corrected for complex sample design

**(P < 0.01)

## Results

### Response and distributions

In the NSAOH a total of n = 14,123 adults responded to the CATI (49% response rate) and n = 5,505 were examined (44% of interviewed people who were invited to the examination). Distributions of explanatory variables are presented in Table 1. The majority of persons attended at a private clinic at the last visit (86.5%). There were slightly higher percentages of females (52.4%) compared to males (47.6%), and check-up (57.0%) compared to problem visits (43.0%). The highest percentage of persons was observed for the $80,000+ income group (31.9%). There were only small percentages of persons in the 75+ year age group (5.7%), reflecting the scope of the study (ie, dentate persons, who had made a dental visit in the last year).

### Unadjusted associations

Services varied by site of last visit for extractions, oral prophylaxis, x-rays, crown/bridge and dentures with private visits associated with low percentages of persons receiving extractions (13.0%) and dentures (2.9%), but high percentages received oral prophylaxis (72.9%) and crown/bridge services (7.0%), as shown in Table 2. Higher percentages of males received extractions (16.2%) compared to females (12.8%), but lower percentages of males received oral prophylaxis services (67.8%) compared to females (72.8%). A higher percentage of persons making problem-oriented visits received fillings (60.5%), extractions (25.9%), x-rays (61.4%), crown/bridge (11.0%), dentures (5.4%) and gum treatment (5.4%) compared to check-up visits (28.1%, 5.8%, 34.8%, 3.1%, 1.6% and 3.6% respectively), while lower percentages of persons making problem visits received oral prophylaxis services (57.7%) compared to check-up visits (80.0%). Income was associated with receipt of fillings, extractions, oral prophylaxis services, x-rays, crown/bridge and dentures. Higher percentages of persons in the high income group received oral prophylaxis, x-ray and crown/bridge services, with lower percentages receiving fillings, extractions and dentures.

### Multivariate models

Multivariate analysis showed that persons attending public clinics had higher odds of extraction, but lower odds of receiving oral prophylaxis and crown/bridge services compared to the reference category of private clinics (Table 3). Males had higher odds of receiving extractions but lower odds of receiving oral prophylaxis services compared to females. Problem-oriented visits were associated with higher odds of receiving fillings, extractions, x-rays, crown/bridge, dentures and gum treatment, but lower odds of receiving oral prophylaxis services compared to check-up visits. Compared to the reference category of low income, higher income groups were associated with higher odds of receipt of fillings, oral prophylaxis, x-ray, and crown/bridge services, but lower odds of receipt of denture services. Compared to the reference category of the youngest age group, older adults had higher odds of receipt of fillings, oral prophylaxis, x-ray, crown/bridge, denture and gum treatment services, but had lower odds for receipt of extractions.

**Table 3:** Multivariate models – % receiving 1+ services by explanatory variables

	**Fillings**		**Extractions**		**Oral prophylaxis**		**X-rays**		**Crown/Bridge**		**Dentures**		**Gum treatment**	
	
	OR	95% CI	OR	95% CI	OR	95% CI	OR	95% CI	OR	95% CI	OR	95% CI	OR	95% CI
**Site last visit^(a)^**														
Public	0.99	(0.76,1.29)	*1.69	(1.26,2.28)	*0.50	(0.38,0.66)	0.97	(0.76,1.24)	*0.34	(0.13,0.91)	1.37	(0.93,2.01)	1.15	(0.65,2.04)
														
**Sex^(b)^**														
Male	1.03	(0.90,1.18)	*1.25	(1.04,1.50)	*0.74	(0.65,0.86)	0.94	(0.82,1.07)	0.94	(0.73,1.19)	0.86	(0.63,1.16)	0.78	(0.58,1.04)
														
**Last visit^(c)^**														
Problem	*3.93	(3.42,4.51)	*5.69	(4.63,6.98)	*0.35	(0.30,0.41)	*3.09	(2.70,3.54)	*3.78	(2.92,4.89)	*2.75	(1.98,3.84)	*1.47	(1.13,1.93)
														
**Income^(d)^**														
$20–<40,000	1.21	(0.98,1.51)	0.81	(0.62,1.05)	*1.39	(1.10,1.77)	*1.32	(1.07,1.63)	*1.73	(1.09,2.73)	*0.57	(0.37,0.86)	0.95	(0.60,1.52)
$40–<60,000	*1.32	(1.04,1.69)	0.75	(0.56,1.01)	*1.57	(1.22,2.02)	*1.61	(1.29,2.01)	*2.19	(1.30,3.70)	*0.48	(0.29,0.80)	1.15	(0.66,2.02)
$60–<80,000	1.26	(0.97,1.63)	0.52	(0.36,0.74)	*1.67	(1.28,2.17)	*1.42	(1.12,1.81)	*1.73	(1.03,2.90)	*0.49	(0.26,0.93)	1.02	(0.57,1.81)
$80,000+	1.12	(0.88,1.42)	0.42	(0.30,0.58)	*2.38	(1.82,3.11)	*1.61	(1.29,2.00)	*2.72	(1.64,4.48)	*0.51	(0.28,0.91)	1.22	(0.73,2.04)
														
**Age^(e)^**														
35–54 years	*1.34	(1.13,1.58)	0.88	(0.68,1.12)	*1.41	(1.18,1.69)	1.02	(0.87,1.20)	*2.09	(1.38,3.18)	*3.01	(1.34,6.76)	*1.69	(1.04,2.72)
55–74 years	*1.51	(1.24,1.83)	*0.75	(0.57,0.98)	*2.01	(1.65,2.44)	0.95	(0.79,1.15)	*3.30	(2.13,5.14)	*6.58	(3.02,14.33)	*2.02	(1.21,3.36)
75+ years	*1.53	(1.12,2.08)	0.72	(0.46,1.13)	*1.96	(1.45,2.65)	*0.70	(0.53,0.93)	*2.93	(1.54,5.60)	*10.19	(4.25,24.46)	1.51	(0.78,2.93)
														
**Pseudo R-squared**	0.149		0.179		0.134		0.101		0.102		0.124		0.017	

Reference categories: (a) Private; (b) Female; (c) Check-up; (d) <$20,000; (e) 15–34 years

*(P < 0.05)

## Discussion

### Representativeness and limitations

Analysis of response patterns and comparisons with Census data revealed that participants differed from non-participants in some characteristics that influence oral health [11]. Participants showed higher percentages who were Australian-born (76.7%), spoke English at home (86.9%) and were employed (64.5%) compared to the Census (69.0%, 80.0% and 55.9% respectively). There was also a higher percentage of non-Indigenous participants (98.5%) and participants with Year 12 schooling level (47.6%) compared to the Census (94.3% and 37.7% respectively). However, some of these differences could reflect higher levels of 'not stated' responses in the Census and differences in coding of some categories for some variables that were compared. When NSAOH estimates of oral health were adjusted to reflect Census distributions of employment, language spoken at home and level of schooling, there were generally small changes suggesting that bias was of a small magnitude. The survey probably underestimated some aspects of oral disease and overestimated the frequency of favourable dental attendance, although the degree of variation was found to be 3% or less for most oral health indicators. Accuracy of survey examiners was assessed by comparison with the survey's principal examiner. The observed levels of agreement for most oral health indicators were equivalent to benchmarks reported for national oral health surveys conducted in the United Kingdom and the United States [11].

The use of self-reported service provision may be considered a limitation of the study. However, the use of dichotomous variables to identify receipt or non-receipt of services in the last year classified in broad categories such as 'filling' is less likely to be reported inaccurately by the general public than, for example, numbers of services received in more specific treatment categories such as 'two-surface amalgam restorations'. A prospective study of the validity of self-reported use of 10 specific types of dental services reported 82% to 100% concordance between self-reports and dental charts [13], with Kappa values of fair to moderate for restorations, moderate for x-rays, dental caps or implants and dentures, and substantial for dental cleaning and for extractions [14]. In addition, since the same unit of service provision is used for both private and public patients it should be comparable. One qualification might be that the disparity observed by site of visit may be underestimated if numbers of services were used instead of receipt versus non-receipt.

### Services provision patterns

The observed service provision patterns show a contrast between public and private dental patients. These partly reflect the problems of resource constraints in the public sector where there is a greater reliance on emergency care that is reflected in a higher percentage of patients receiving extractions and a lower percentage receiving preventive and maintenance services. These service patterns persisted when controlled for the effects of age and sex, which have been reported to impact on dental service profiles [9,10].

In addition, the impact of problem visits and income persisted as independent effects. Previous reports have shown that regular attendance has a positive impact on oral health [15], and that irregular, problem-oriented visiting is related to poorer oral health such as fewer teeth [16]. Many studies have shown inequalities in oral health and services received, such as children from deprived backgrounds being more likely to receive extractions compared to their more affluent peers [17]. However, differences in oral health by payment method were found to be largely attributable to socio-demographic factors and regularity of dental attendance rather then method of payment itself [18]. Increasing levels of socio-economic disadvantage have been related to worse oral health and decreased utilisation of services [19].

Extraction of teeth is the dental equivalent of mortality and reflects attitudes of patients and providers, availability and accessibility of care, and philosophies of dental treatment [20]. Patterns of tooth loss by social class have been linked not only to cultural factors such as attitudes but also to the delivery system itself [21]. When taken together with the high levels of emergency visits in public dental care, this indicates a service pattern that is in contrast to minimum intervention philosophy for dental care [22], in which dentists aim to provide a high frequency of monitoring and preventive care, but low frequency of restoration and extraction. This is most likely a reflection of access problems such as long waiting times for public patients. Having to wait for extended periods of time on waiting lists for general care may result in deterioration in oral health so that when they eventually attend for care their condition is more severe than when care was initially sought, or their oral health may deteriorate to the point where they seek emergency care for relief of pain. The high level of emergency care among public patients restricts the treatment options that are available and may lead both providers and patients to opt for rapid resolution of the problem through extraction of teeth. Recent research has revealed that the dental visiting behaviour of adult public dental patients was influenced by limitations of the public dental system. Patients appeared to be using emergency dental care services (which increased their likelihood of receiving extractions) because of lack of access to general dental care services. It was found that patient's attitudes toward their dental health and dental visiting were fairly positive, but the experience of structural barriers such as cost of dental treatment and long waiting lists prevented them from receiving the care they need or would like. This finding moves beyond the individual and recognises that the system plays a vital role in the lack of access to much needed services. Unless structural barriers to dental care are addressed, patients will engage in dental visiting behaviours that place them at risk of worse oral health outcomes, and this pattern of behaviour could be perpetuated indefinitely [23].

It has been observed that public funding for dental care in Australia favours the financially and orally better off at the expense of disadvantaged and orally unhealthy Australians [8]. Other countries that have dental care systems that are predominantly private sector oriented such as the USA note that there is a growing disconnect between the dominant pattern of practice and the oral health needs of the nation [24]. In the USA reducing health disparities, including oral health disparities is a major goal for public and private health agencies [25]. In Canada an increasing reliance on private funds has been linked to greater barriers to care, particularly among less affluent groups [26]. European union countries show wide variation in their oral health care provision systems, but there has been privatisation of many previously public dental services [27]. The findings of the present study show that the same problems faced by card holders and public dental services in the 1990s that led to a government funded Commonwealth Dental Health Program in the 1990s have not gone away some decade later.

## Conclusion

Socio-economically disadvantaged persons who face barriers to accessing dental care in the private sector suffer further oral health disadvantage from a pattern of services received at public clinics that has more emphasis on extraction of teeth and less emphasis on preventive and maintenance care.

## Competing interests

The author(s) declare that they have no competing interests.

## Authors' contributions

DSB, LL and KR-T conceived of the research problem. DSB and LL planned and conducted the analysis. DSB drafted the manuscript, and LL and KR-T participated in completing the manuscript. All authors read and approved the manuscript.

## Pre-publication history

The pre-publication history for this paper can be accessed here:


